# TGFβ signalling plays an important role in IL4-induced alternative activation of microglia

**DOI:** 10.1186/1742-2094-9-210

**Published:** 2012-09-04

**Authors:** Xiaolai Zhou, Björn Spittau, Kerstin Krieglstein

**Affiliations:** 1 Institute for Anatomy and Cell Biology, Department of Molecular Embryology, Albert-Ludwigs-University Freiburg, Albertstraße 17, Freiburg 79104, Germany; 2 Faculty of Biology, Albert-Ludwigs-University Freiburg, Hauptstraße 1, 79104 Freiburg, Germany; 3 Freiburg Institute for Advanced Studies (FRIAS), Albert-Ludwigs-University Freiburg, Albertstraße 19, Freiburg 79104, Germany

**Keywords:** Microglia, TGFβ, IL4, Alternative activation, Arg1, Ym1

## Abstract

**Background:**

Microglia are the resident immune cells of the central nervous system and are accepted to be involved in a variety of neurodegenerative diseases. Several studies have demonstrated that microglia, like peripheral macrophages, exhibit two entirely different functional activation states, referred to as classical (M1) and alternative (M2) activation. TGFβ is one of the most important anti-inflammatory cytokines and its effect on inhibiting microglia or macrophage classical activation has been extensively studied. However, the role of TGFβ during alternative activation of microglia has not been described yet.

**Methods:**

To investigate the role of TGFβ in IL4-induced microglia alternative activation, both, BV2 as well as primary microglia from new born C57BL/6 mice were used. Quantitative RT-PCR and western blots were performed to detect mRNA and protein levels of the alternative activation markers Arginase1 (Arg1) and Chitinase 3-like 3 (Ym1) after treatment with IL4, TGFβ or both. Endogenous TGFβ release after IL4 treatment was evaluated using the mink lung epithelial cell (MLEC) assay and a direct TGFβ2 ELISA. TGFβ receptor type I inhibitor and MAPK inhibitor were applied to address the involvement of TGFβ signalling and MAPK signalling in IL4-induced alternative activation of microglia.

**Results:**

TGFβ enhances IL4-induced microglia alternative activation by strongly increasing the expression of Arg1 and Ym1. This synergistic effect on Arg1 induction is almost completely blocked by the application of the MAPK inhibitor, PD98059. Further, treatment of primary microglia with IL4 increased the expression and secretion of TGFβ2, suggesting an involvement of endogenous TGFβ in IL4-mediated microglia activation process. Moreover, IL4-mediated induction of Arg1 and Ym1 is impaired after blocking the TGFβ receptor I indicating that IL4-induced microglia alternative activation is dependent on active TGFβ signalling. Interestingly, treatment of primary microglia with TGFβ alone results in up regulation of the IL4 receptor alpha, indicating that TGFβ increases the sensitivity of microglia for IL4 signals.

**Conclusions:**

Taken together, our data reveal a new role for TGFβ during IL4-induced alternative activation of microglia and consolidate the essential functions of TGFβ as an anti-inflammatory molecule and immunoregulatory factor for microglia.

## Background

Microglia represent the resident immune cells of the central nervous system (CNS) and account for approximately 12% of all cells in the brain [1]. As counterparts of peripheral macrophages, microglia sense the brain parenchyma for perturbations resulting from injury or pathological conditions. Several CNS neurodegenerative pathologies including Alzheimer’s disease (AD) [2-4], multiple sclerosis (MS) [5,6] and Parkinson’s disease (PD) [7,8] are characterised by a strong microglia reaction that is, at least partially, responsible for the progressive nature of these diseases.

Increasingly, studies have demonstrated that microglia, like peripheral macrophages, exhibit two entirely functional different activation states that are referred to as classical and alternative activation. The classical activation of microglia (M1) is induced by Th1 cytokines, such as IFNγ, IL1β, IL12, and IL6 as well as lipopolysaccharide (LPS) and results in production and release of pro-inflammatory cytokines such as tumour necrosis factor-alpha (TNFα), IL6, matrix metalloproteinase (MMP)-9, nitric oxide (NO) and reactive oxygen/nitrogen species (ROS) [9-11] which are involved in inflammation-mediated neurotoxicity [12-14]. Whereas alternative activation of microglia (M2) initiated by Th2 cytokines, such as IL4 and IL13, as well as IL10 and TGFβ, results in up regulation of arginase-1 (Arg1), Chitinase 3 like 3 (Ym1) and found in inflammatory zone-1 (Fizz1), which are primarily associated with tissue repair and extracellular matrix composition [3,11]. As hypothesised by Town *et al.* these differently activated microglia likely exist as a dynamic continuum *in vivo*, with functions ranging from deleterious to beneficial [15,16]. All these notions suggest that modulating microglia activation states might be a potential therapeutic approach to different types of neurodegenerative diseases including AD, MS, and PD.

Based on the M1 and M2 activation states, a more detailed categorization of macrophage activation states has recently been discussed. As suggested by Gordon and colleagues [11,17,18], alternative activation is only limited to macrophages treated with IL4 or IL13 and is primarily associated with injury resolution including tissue repair and extracellular matrix reconstruction. While IL10 and TGFβ promote a macrophage phenotype characterised by inflammation resolution including inhibition of pro-inflammatory cytokine production, modification of inflammatory signalling pathways and increased expression of scavenger receptors, thereby, promoting debris clearance. This type of activated macrophage has been termed acquired deactivation [19-23]. Whereas IL10 and TGFβ induce acquired deactivation, the acquired deactivation macrophages can further produce IL10 and TGFβ in an autocrine manner [3,24,25]. Regarding the immunoregulatory function of IL10 and TGFβ produced by the acquired deactivation macrophages, this phenotype of macrophages has been also named as a regulatory macrophage by Mosser and Edwards [24]. Although these studies promoted the knowledge of microglia/macrophage activation phenotypes and their distinct functions, there is still no general agreement in the field on the nomenclature and, more importantly, the interaction between different activation states of microglia/macrophages is still poorly understood.

TGFβ is a multifunctional cytokine involved in a variety of physiological and pathological conditions [26]. TGFβs bind to the TGFβ receptor type II, which recruits and phosphorylates a type I receptor. The type I receptor then phosphorylates Smad2/3, which further bind to Smad4 to form a heteromeric complex that translocates into the nucleus to regulate the expression of target genes [27,28]. Next to the canonical Smad-dependent pathway, TGFβs also signal via Smad-independent signalling cascades, including mitogen-activated protein kinase signalling (MAPK) pathways [29].

In this study we used the microglial cell line BV2 and primary microglia to investigate the role of TGFβ in IL4-induced alternative activation, thereby illustrating the interaction between different microglia/macrophage activation states. For the first time, we provide evidence that although TGFβ1 treatment alone is not able to induce microglia, alternative activation, treated together with IL-4, strongly enhances IL4-induced alternative microglia activation. Arg1 and Ym1 expression was significantly increased after co-treatment with IL4 and TGFβ1. To our surprise, Arg1 and Ym1 expression induced by IL4 treatment alone was significantly impaired in the presence of the TGFβ receptor type I inhibitor. Further investigation revealed that IL4 treatment alone increased microglial TGFβ2 expression and secretion, which in turn might promote IL4-induced Arg1 and Ym1 expression. Moreover, we found TGFβ1 treatment resulted in up regulation of the IL4 receptor alpha (IL4Rα). Finally, we provide evidence that the Mitogen-activated protein kinase (MAPK) pathway is essential for TGFβ-mediated enhancement of Arg1 expression after IL4 treatment in microglia.

## Methods

### Cytokines and reagents

All reagents for cell culture, namely Trypsin-EDTA 1×, Hank’s balanced salt solution (BSS), Dubecco’s modified Eagle medium (DMEM)-Ham’s F12, penicillin/streptomycin (P/S) 100×, and fetal calf serum (FCS) were purchased from PAA Laboratories (Cölbe, Germany). MAPK/ERK (MEK) inhibitor PD98059 and poly-D-lysine were purchased from Sigma-Aldrich (Deisenhofen, Germany). Recombinant murine IL4 and recombinant human TGFβ1 were purchased from PeproTech (Hamburg, Germany). TGFβ receptor type I kinase inhibitor (TβKI) was obtained from Merck Chemicals (Darmstadt, Germany). Primary antibodies: anti-Arginase1, anti-IL4Rα, anti-TGFβ2 and anti-Smad1/2/3 were purchased from SantaCruz (Heidelberg, Germany). Phospho-Smad2-Ser465/467 and phospho-Stat6-Tyr641 were obtained from New England Biolabs (Frankfurt, Germany), Ym1 antibody was from StemCell Technologies (Grenoble, France). GAPDH was purchased from Abcam (Cambridge, UK). Goat anti-mouse Cy3, goat anti-rabbit Cy3 were from Dianova (Hamburg, Germany).

### BV2 cell culture

The murine microglia cell line BV2 was maintained in DMEM/F12 (PAA) supplemented with 10% heat-inactivated FCS and 1% P/S. Cultures were kept at 37°C in 5% CO_2_/95% humidified air atmosphere. Prior to treatment cells were washed with PBS and serum-free medium was added.

### Primary microglia cultures

Whole brains obtained from P0/1 C57BL/6 mice were washed twice with Hank’s BSS solution and vessels and meninges were removed from brain surfaces under the microscope. Cleaned brains were collected and enzymatically dissociated with Trypsin-EDTA (1×) for 15 minutes at 37°C. An equal amount of ice-cold FCS, together with DNase I (Roche Diagnostics, Mannheim, Germany) at a final concentration of 0.5 mg/ml was added prior to dissociation with wide- and narrow-bored polished Pasteur pipettes. Cells were then washed and single cells were centrifuged, collected and suspended with (DMEM)-Ham’s F12 medium containing 10% fetal bovine serum (FBS) and 1% Penicillin/Streptomycin. Cell suspensions were transferred to poly-D-lysine-coated tissue culture flasks with a density of 2 brains/75 cm^2^ flask. Cultures were maintained in a humidified 5% CO_2_/95% air atmosphere at 37°C. At day *in vitro* (DIV) 2 and 3, cultures were washed twice with pre-warmed phosphate- buffered saline (PBS) and fresh culture medium was added. After 10 to 14 days in culture, microglia were shaken off from adhesive grown astroglia by shaking at approximately 250 to 300 rpm for 1 hour. Isolated microglia were plated into various dishes or plates and treated with proper factors, according to different experimental purposes.

### Immunocytochemistry

Microglia were plated on glass coverslips and were fixed after treatment with 4% paraformaldehyde (PFA) for 15 minutes at room temperature. After blocking with PBS containing 10% normal goat serum and 0.1% TritonX-100 (Roche, Mannheim, Germany) for 1 hour at room temperature, cells were incubated with primary antibodies at 4°C overnight, followed by an incubation with corresponding Cy3-conjugated secondary antibodies (goat anti-mouse Cy3 1:100, goat anti-rabbit Cy3 1:100). Nuclei were counterstained using 4′,6-diamidino-2-phenylindole (DAPI, Roche). Phase contrast and fluorescence images were captured using the Leica AF6000 imaging system (LEICA, Wetzlar, Germany).

### Protein isolation and western blotting

Total proteins were isolated from primary microglia and BV2 cells after washing with PBS and incubation with ice-cold mammalian protein extraction reagent (M-PER, Thermo Scientific, Bonn, Germany) plus Complete Protease Inhibitor (Roche) with gentle up and down shaking for 5 minutes. The supernatant as well as debris were collected, and centrifuged at 14,000 rpm for 8 minutes to obtain the supernatant, which contains proteins. After determination of protein concentrations, 10–15 μg total proteins were loaded onto 10 to 12% SDS gels. After electrophoresis, proteins were transferred to polyvinylidene difluoride (PVDF) membranes (Immobilon, Milipore, Schwalbach, Germany). Blots were blocked with 5% non-fat dry milk in Tris-buffered saline containing 0.05% Tween-20 (TBST) for 1 h and incubated with primary antibodies overnight at 4°C. Primary antibodies against Arginase-1 (rabbit polyclonal, 1:1000, SantaCruz), phospho-Smad2 (Ser465/467) (rabbit, 1:1000, Cell Signaling), phospho-Stat6 (Tyr641) (rabbit, 1:1000, Cell Signaling), IL4RÎ± (mouse, 1:1000, SantaCruz), TGFβ2 (rabbit polyclonal, 1:1000, SantaCruz), Ym1 (rabbit polyclonal, 1:1000, StemCell Technologies) and glyceraldehyde-3-phosphate dehydrogenase (GAPDH) (mouse monoclonal, 1:10,000, Abcam, Cambridge, UK) were used. After incubation with goat anti-rabbit or goat anti-mouse IgG horseradish peroxidase (HRP)-linked antibodies (1:10,000, Cell Signaling), blots were developed using Western Lightning® Plus-ECL, Enhanced Chemiluminescence Substrate (Perkin-Elmer, Rodgau, Germany). Signals were captured on Amersham Hyperfilm™ECL (GE Healthcare, München, Germany). Band intensities were evaluated using the software FlourChem 8800 (Alpha Innotech, Biozym, Olendorf, Germany).

### RNA isolation and quantitative RT-PCR

RNA was isolated from BV2 and primary microglial cells with the RNeasy kit (Qiagen, Hilden, Germany), according to the manufacturer’s instructions. RNA was reverse transcribed to cDNA with the GeneAmp RNA PCR Core Kit (Applied Biosystems, Darmstadt, Germany). Quantitative RT-PCR (qRT-PCR) analysis was performed with the MyiQ™ (BIO-RAD, München, Germany) and the Quantitect SYBR Green PCR Kit (Applied Biosystems) with 1 μl of cDNA template in a 25 μl reaction mixture. Results were analysed with the Bio-Rad iQ5 Opitcal System Software and the comparative CT method. Data are expressed as 2^-ΔΔCT^ for the experimental gene of interest normalized to the housekeeping gene (GAPDH) and presented as fold change relative to control. The following primers were used: TGFβ1for: 5^′^-TAATGGTGGACCGCAACAACG-3; TGFβ1rev: 5^′^-TCCCGAATGTCTGACGTATTGAAG-3 [NM_011577.1, NCBI]; TGFβ2for: 5^′^-AGAATCGTCCGCTTTGATGTCTC-3^′^, TGFβ2rev: 5^′^-ATACAGTTCAATCCGCTGCTCG-3^′^ [NM_009367.3, NCBI]; TGFβ3for: 5^′^-GCCCTGGACACCAATTACTGC-3; TGFβ3rev: 5^′^-CCTTAGGTTCGTGGACCCATTTC-3´ [NM_009368.3, NCBI]; Arg1for: 5^′^-TCATGGAAGTGAACCCAACTCTTG-3^′^, Arg1rev: 5^′^-TCAGTCCCTGGCTTATGGTTACC-3^′^ [NM_007482.3, NCBI]; Ym1for: 5^′^-AGACTTGCGTGACTATGAAGCATTG-3^′^; Ym1rev: 5^′^-GCAGGTCCAAACTTCCATCCTC-3^′^ [NM_009892.2, NCBI]; IL4for: 5^′^-ATTTTGAACGAGGTCACAGGAGAAG-3^′^; IL4rev: 5^′^-ACCTTGGAAGCCCTACAGACGAG-3^′^ [NM_021283.2, NCBI]; IL4Rαfor: 5^′^-GAACTCAGACCCACCCAAAAGC-3^′^; IL4Rαrev: 5^′^-AAGTGGCAAGTGAGGGACGAG-3^′^ [NM_001008700.3, NCBI]; Gapdhfor: 5^′^-ATGACTCTACCCACGGCAAG-3^′^; Gapdhrev: 5^′^-GATCTCGCTCCTGGAAGATG-3^′^ [NM_008084.2, NCBI].

### Characterisation of TGFβ secretion

Primary microglia were treated with or without IL4 (10 ng/ml) in serum-free DMEM-Ham’s F12 medium for 24 hours. Conditioned medium was collected for the mink lung epithelial cell (MLEC) assay and ELISA. The MLEC assay is widely used to measure the amount of TGFβs in conditioned medium. The principal is that the MLECs containing a luciferase reporter under the control of a TGFβ-responsive truncated plasminogen activator inhibitor (PAI)-promoter are able to generate luciferase with a TGFβs dose-dependent manner. Since the MLECs only response to the activated TGFβs, in order to detect the latent part of TGFβs, the conditioned medium has to be acidification first to convert latent TGFβs into activated ones. To evaluate the levels of released TGFβs after IL4 treatment, the MLEC assay was performed as described by Abe *et al.*[30]. Briefly, MLECs were placed into 96-well plates at the density of 1.5 × 10^4^ cells per well and treated with collected conditioned medium either with or without acidification with 1 M HCL and pH adjustment with NaOH (to activate latent TGFβs) as well as the standard mediums containing different contractions of recombinant TGFβ for 16 hours. Cells were washed with PBS and total proteins were extracted using lysis buffer (Tropix, Applied Biosystems). The luciferase activity was analysed in duplicates using a luminometer (LumatB5076, Berthold, Bad Wildbad, Germany).

### Direct enzyme-linked immunosorbent assay for TGFβ2 detection

Conditioned media collected from primary microglia after treatment with and without IL4 (10 ng/ml), as well as diluted recombinant human TGFβ2 (2000, 1000, 500, 250, 125, 62.5 and 31.25 pg, R&D Systems, Wiesbaden-Nordenstedt, Germany) standard solutions, were added into ELISA plates (NUNC, Wiesbaden, Germany) and incubated overnight at 4°C. After washing with washing buffer (0.05% Tween-20 in PBS) and blocking with 1% BSA in PBS for 2 hours at 37°C, the plates were incubated with anti-TGFβ2 (Santa Cruz) primary antibodies overnight at 4°C. Followed by washing and incubation with Biotin-linked anti-rabbit secondary antibodies for 2 hours at 37°C, plates were incubated with ABC-solution (Vectashield, BIOZOL, Eching, Germany). Colour reaction was performed using 2, 2^′^-azino-bis (3-ethylbenzothiazoline-6-sulphonic acid) substrate (ABTS, Sigma-Aldrich, Deisenhofen, Germany) for 30 minutes in the dark. Finally, absorbance was detected using an FC Multiskan plate reader (Thermo Fischer, Bonn, Germany) at the absorption of 405 nm.

### Cytokine array

For the analysis of IL4 release, supernatant from untreated and TGFβ1-treated primary microglia was analysed using the Proteome Profiler™ Array Mouse Cytokine Array Panel A (R&D Systems, Wiesbaden-Nordenstedt, Germany) according to the manufacturer’s instructions. Briefly, equal amounts of primary miroglia cells were incubated for 24 hours in the presence or absence of TGFβ1 and media were collected. Cytokine array membranes were incubated with cell culture supernatants at 4°C overnight with gentle shaking. Membrane signals were developed using Western Lightning® Plus-ECL, Enhanced Chemiluminescence Substrate (Perkin-Elmer, Germany) and signals were captured on Amersham Hyperfilm™ ECL (GE Healthcare).

### Statistical procedures

The data were expressed as means ± standard error (SE). Statistical significance between multiple groups was compared by one-way analysis of variance (ANOVA) followed by an appropriate multiple comparison test. Two-group analysis was performed using the Student’s *t*-test. *P*-Values < 0.05 were considered statistically significant. All statistical analyses were performed using GraphPad Prism4 software (GraphPad Software Inc.).

## Results

### TGFβ1 enhances IL4-induced alternative microglia activation

**Figure 1:**
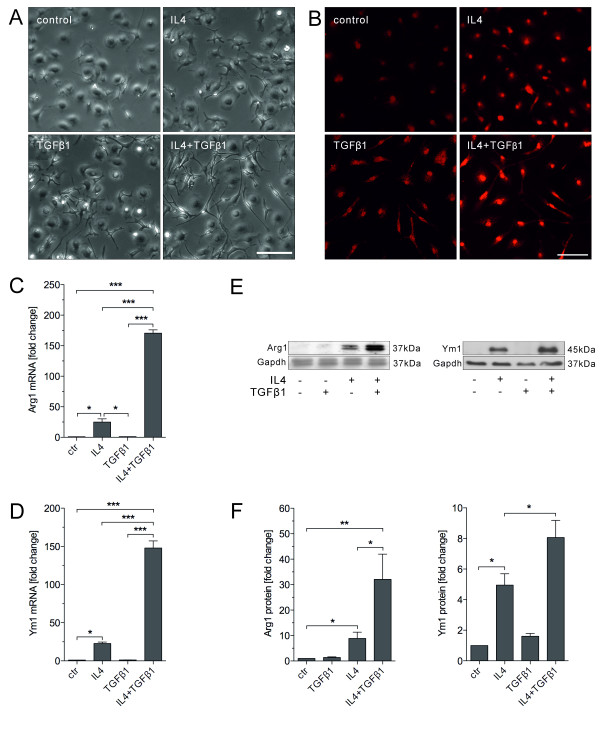
**TGFβ1 enhances IL4-induced alternative activation of microglia.** (**A**) Primary cultured microglial cells changed their morphology from round-shaped into ramified after treated with IL4 (10 ng/ml) and TGFβ1 (1 ng/ml) for 24 hours. This cellular morphological change was enhanced when microglia were co-treated with IL4 and TGFβ1. (**B**) Immunofluorescence staining for Arg1 demonstrated increased staining intensity for Arg1 after treatment with IL4 or TGFβ alone. Combined treatment with IL4 and TGFβ1 strongly enhanced Arg1 immunoreactivity. Scale bars indicate 100 μm. Quantitative RT-PCR showed increased Arg1 (**C**) and Ym1 (**D**) mRNA levels in primary microglia after IL4 treatment. Co-treatment with IL4 and TGFβ1 for 24 hours significantly increased Arg1 and Ym1 mRNA levels in primary microglia. (**E**) Western blotting revealed increased Arg1 and Ym1 protein levels in primary microglia after treatment with IL4. Again, co-treatment with IL4 and TGFβ1 increased the protein levels of Arg1 and Ym1. Representative western blot results from at least three independent experiments are shown. GAPDH was used as control for equal protein loading. (**F**) Densitometric evaluation of Arg1 and Ym1 band intensities and statistical analysis. Data are given as means ± standard error from three independent experiments: *P < 0.05, **P < 0.01, ***P < 0.001 (one-way analysis of variance).

To investigate the influence of TGFβ1 on IL4-induced microglia-alternative activation, primary microglia were treated either with IL4 (10 ng/ml), TGFβ1 (1n g/ml) or with a combination of both factors for 24 hours. As a crude readout for microglia activation, the morphology change of microglia was analysed after treatment. Treatment with IL4 or TGFβ1 alone for 24 hours resulted in morphology changes in BV2 cells (data not shown) and primary microglia towards a more ramified phenotype. This extent of morphological change was remarkably increased when the cells were treated with IL4 and TGFβ1 together (Figure 1A). As the morphological change could not always precisely reflect the activation states, the assessment of the alternative activation still relied on the molecule markers such as Arg1 and Ym1. Therefore, the expression of Arg1 and Ym1 were analysed. Immunofluorescence staining demonstrated increased Arg1 staining intensity after IL4 treatment. Combination of IL4 and TGFβ1 further increased the staining intensity (Figure 1B). Using quantitative RT-PCR the up regulation of Arg1 and Ym1 was determined. A significant increase in Arg1 and Ym1 RNA levels was observed after treatment with IL4 alone. TGFβ1 treatment alone did not result in increased Arg1 and Ym1 mRNA levels (*P* > 0.05). However, treatment with IL4 and TGFβ1 resulted in significant increase of Arg1 and Ym1 RNA levels (*P* < 0.001) compared to IL4 treatment alone (Figure 1C, D). As shown in Figure 1E and F, IL4 treatment significantly increased Arg1 and Ym1 protein levels in primary microglia (*P* < 0.05). TGFβ1 slightly increased Arg1 and Ym1 protein levels in primary microglia, without reaching significant differences compared to control (*P* > 0.05). Combination of IL4 and TGFβ1 significantly increased IL4-induced Arg1 and Ym1 protein levels in primary microglia (*P* < 0.05).

### IL4-induced Arg1 and Ym1 upregulation is dependent on TGFβ signalling

**Figure 2:**
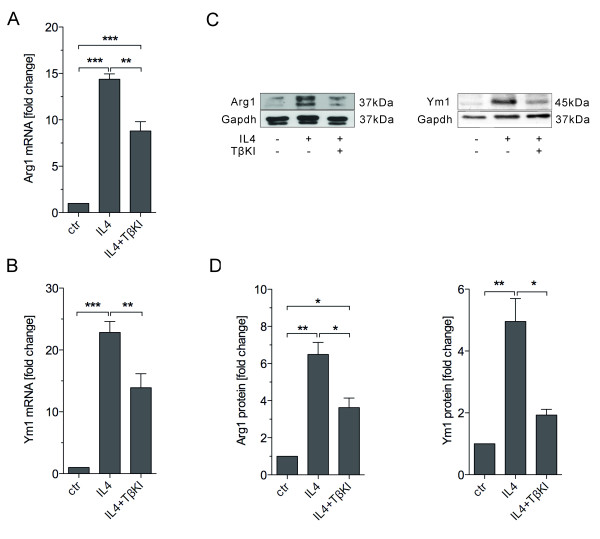
**Arg1 and Ym1 expression induced by IL4 was blocked in the presence of a TGFβ receptor type I inhibitor.** Primary microglia were treated with IL4 (10 ng/ml) combined either with or without TGFβ receptor type I kinase inhibitor IV (TβKI, 2 μM) for 24 hours. RNA and proteins were isolated for quantitative RT-PCR and western blotting, respectively. Quantitative RT-PCR shows that IL4 treatment significantly increased Arg1 (**A**) and Ym1 (**B**) mRNA levels (*P* < 0.001) which was partially blocked by co-treatment with TβKI (*P* < 0.01). Western blotting (**C**) shows Arg1 and Ym1 protein levels in primary microglia after different treatments, which were quantified by densitometric analysis, and normalized to Gapdh (**D**). IL4 treatment significantly increased Arg1 and Ym1 protein levels in primary microglia (*P* < 0.01), which was significantly blocked by TβKI in microglia (*P* < 0.05). Data are presented as mean ± standard error from three independent experiments: *P < 0.05, **P < 0.01, ***P < 0.001 (one-way analysis of variance).

In order to address whether endogenous TGFβ signalling is involved in IL4-induced alternative microglia activation, primary microglia were treated with the combination of IL4 and TGFβ type I receptor kinase inhibitor IV (TβKI). We found expression of Arg1 and Ym1 induced by IL4 were partially impaired by TβKI. As is shown in Figure 2, primary microglia were treated either with IL4 (10 ng/ml) or IL4 (10 ng/ml) together with TβKI (2 μM/ml) for 24 hours, there the mRNA and protein were isolated for qRT-PCR and western blotting, respectively. qRT-PCR revealed that Arg1 and Ym1 mRNA up regulation after IL4 treatment was significantly reduced by co-treatment with TβKI (Figure 2A, B). Western blotting results demonstrate that Arg1 and Ym1 protein levels in primary microglia were increased after IL4 treatment and significantly decreased in the presence of TβKI (Figure 2C, D). Similar results have been achieved from BV2 cells (data not shown). All these data indicate that IL4-induced Arg1 and Ym1 expression is at least partially dependent on endogenous TGFβ signalling in microglia after IL4 treatment.

### IL4-treated microglia increase TGFβ2 expression and secretion

**Figure 3:**
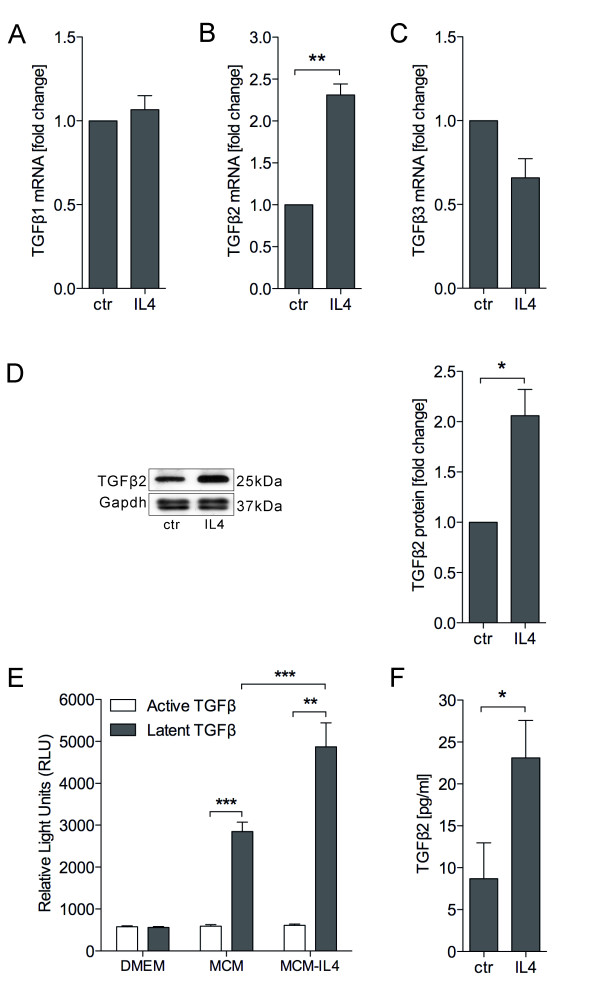
**Treatment of microglia with IL4 increased TGFβ2 expression and secretion.** Primary microglia were treated with or without IL4 (10 ng/ml) for 24 hours. Total mRNA and proteins were isolated from the cells for RT-PCR and western blotting, respectively. Conditioned medium from IL4-treated microglia (MCM-IL4) as well as non-treated microglia (MCM) was collected and the mink lung epithelial cell (MLEC) assay and enzyme-linked immunosorbent assay (ELISA) were performed. Quantitative RT-PCR for TGFβ1 (**A**), TGFβ2 (**B**) and TGFβ3 (**C**) revealed increased TGFβ2 expression after IL4 treatment. Intracellular TGFβ2 protein levels were significantly increased (*P* < 0.05) in primary microglia after treatment with IL4 (**D**). MLEC assay (**E**) shows that primary microglia secreted a certain amount of inactive TGFβ, which was significantly increased by IL4 treatment (*P* < 0.01). Direct TGFβ2 ELISA (**F**) showed that TGFβ2 secretion was significantly increased after IL4 treatment (*P* < 0.05). All experiments were repeated at least three times. Data are presented as mean ± standard error: *P < 0.05, **P < 0.01, ***P < 0.001(Student’s *t*-test).

To investigate the endogenous TGFβs expression and secretion form microglia after IL4 treatment, primary microglia were treated with or without IL4 (10 ng/ml) for 24 hours. The cells were harvested for mRNA extraction and qRT-PCRs for different isoforms of TGFβ were performed. Quantitative RT-PCR demonstrates that among all TGFβ isoforms, only TGFβ2 mRNA was significantly up regulated after IL4 treatment (Figure 3A,B,C). Since the TGFβ receptor inhibitor used above is not specific for TGFβ1, 2 or 3, in addition to the blockage of TGFβ1, 2, 3, it also inhibits Activin and Nodal signalling. Therefore, the mRNA levels of the Activin A, Activin B, and Nodal were also analysed using qRT-PCR but were not changed after IL4 treatment (data not shown). The protein levels of intracellular TGFβ2 were significantly increased (*P* = 0.028) after treatment with IL4 (Figure 3D). Since endogenous TGFβ2 in primary microglial cells is up regulated after IL4 treatment, we further addressed whether TGFβ secretion from IL4-treated microglia is also increased. Therefore, the conditioned media from IL4-treated (MCM-IL4) as well as non-treated microglial cells (MCM) were harvested after 24 hours and the MLEC assay was performed to monitor TGFβ secretion. Quantification of TGFβ-induced intensity of luciferase shows that primary microglia secreted TGFβ under basal conditions and most of this was in a latent and inactive state. IL4 treatment significantly increased latent TGFβ secretion (Figure 3E). Since the MLEC assay is not specific for different TGFβ isoforms, based on the qRT-PCR results we used a direct ELISA for TGFβ2 and demonstrated a significant increase in TGFβ2 secretion after IL4 treatment (Figure 3F).

**Figure 4:**
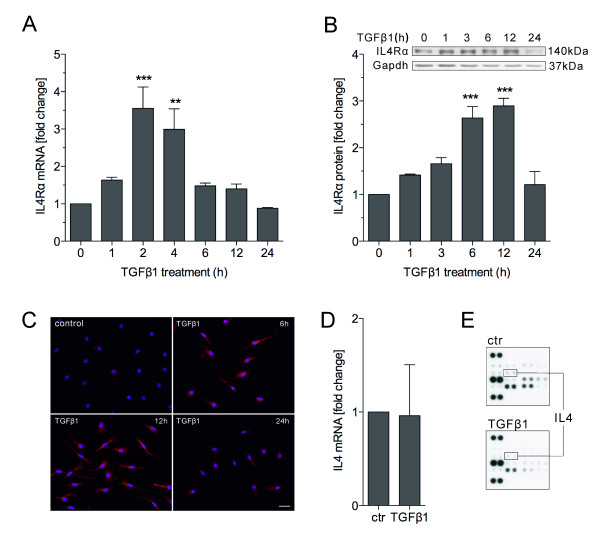
**TGFβ1 up regulates the IL4Rα.** Primary microglia were treated with TGFβ1 (1 ng/ml) for different time points and the cells were either harvested for analysing IL4Rα mRNA and protein levels, or fixed with 4% paraformaldehyde (PFA) for IL4Rα immunostaining. Quantitative RT-PCR showed that treatment with TGFβ1 increased IL4Rα mRNA levels starting after 1 hour and peaking at around 2 hours. Afterwards mRNA levels decreased again and returned to basal levels at 24 hours (**A**). Western blotting showed IL4Rα protein expression starting to increase after treatment with TGFβ1 for 1 hour and peaking at around 6 to 12 hours after treatment (**B**). After treatment with TGFβ1 for 6 and 12 hours, IL4Rα immunoreactivity (red) was increased and cell morphology changed towards a ramified phenotype compared to control cells. After 24 hours, the IL4Rα immunoreactivity (red) was decreased but the cells still presented a ramified shape with long processes (**C**). Scale represents 20 μm. Treatment of primary microglia with TGFβ1 (n = 5) had no effect on IL4 mRNA levels (**D**). Analysis of TGFβ1-mediated changes in microglial cytokine release (n = 2) demonstrated no differences in IL4 levels after TGFβ1 treatment (**E**). Data (**A**,**B, D**) are presented as means ± standard error: **P < 0.01, ***P < 0.001 (one-way analysis of variance).

#### TGFβ1 increases IL4Rα expression in primary microglia

Based on our observation that TGFβ1 also enhances the IL13-induced Arg1 up regulation in BV2 cells and primary microglia (data not shown), as well as the knowledge that IL4 and IL13 share the IL4Rα as a common receptor, which promotes phosphorylation of the transcription factor Stat6 that finally induces Arg1 expression [11,31], we analysed whether IL4Rα is regulated by TGFβ1. Primary microglia were treated with TGFβ1 (1 ng/ml) for different time points and RNA and proteins were isolated. The qRT-PCR results demonstrate that TGFβ1 treatment significantly increased the mRNA levels of IL4Rα after 2 and 4 hours, with the peak at 2 hours. From 6 to 24 hours the levels decreased and finally returned to basal levels at 24 hours after TGFβ1 treatment (Figure 4A). Western blotting confirmed the TGFβ1-mediated up regulation of IL4Rα. IL4Rα protein levels increased after treatment with TGFβ1 reaching the maximum from 6 to 12 hours. After treatment for 24 hours, IL4Rα protein levels returned to basal levels (Figure 4B). Immunostaining for IL4Rα after treatment with TGFβ1 for 6, 12 and 24 hours showed a similar pattern. IL4Rα staining intensity was increased 6 and 12 hours after treatment with TGFβ1. After 24 hours, the IL4Rα signal was comparable to the control condition (Figure 4C). We further analysed whether TGFβ1 has an effect on microglial IL4 expression and release. As shown in Figure 4D and E, IL4 mRNA levels were not significantly changed after TGFβ1 treatment for 24 hours. Using a mouse-specific cytokine array, we revealed that primary microglia release very low levels of IL4 and treatment of primary microglia with TGFβ1 did not result in any changes in IL4 release after 24 hours. These data suggest that the enhancement of Arg1 and Ym1 expression by TGFβ in IL4-treated microglia might, at least partially, be mediated by increasing IL4Rα expression, thus, enhancing the microglial sensitivity to IL4 signals.

#### Mitogen-activated protein kinase mediates TGFβ1-enhanced Arg1 expression in IL4-treated primary microglia

**Figure 5:**
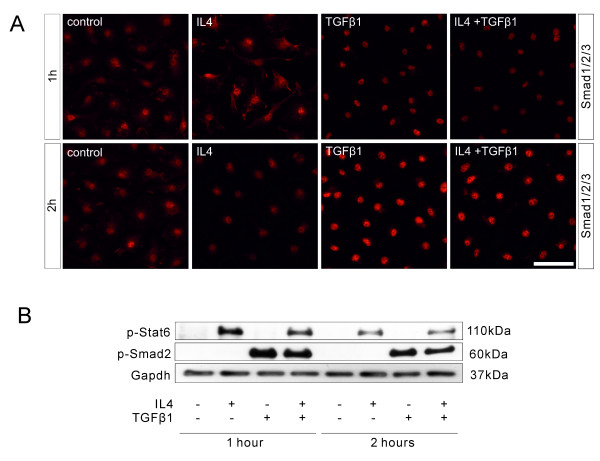
**Direct interactions were not observed between the TGFβ/Smad signalling pathway and the IL4/Stat6 signalling pathways.** Primary microglial cells were treated with either with IL4 (10 ng/ml), TGFβ1 (1 ng/ml) alone or together for 1 and 2 hours. The cells were either fixed with 4% paraformaldehyde (PFA) for pSmad2/3 immunostaining or harvested for testing pStat6 and pSmad2 levels. The treatment of TGFβ1 alone or TGFβ1 combining IL4-induced pSmad2/3 nuclear translocation both at 1 and 2 hours while IL4 treatment alone was not able to induced pSmad2/3 nuclear translocation (**A**). Western blotting showed that IL4 treatment alone exclusively induced Stat6 phosphorylation but not Smad2 phosphorylation after 1 and 2 hours, while TGFβ1 treatment alone or TGFβ1 in combination with IL4 only, induced Smad2 phosphorylation but not Stat6 phosphorylation (**B**).

To investigate the pathways involved in TGFβ1-mediated enhancement of IL4-induced Arg1 expression, the TGFβ/Smad and the IL4/Stat6 signalling pathways were analysed by monitoring Smad2/3 nuclear accumulation and phosphorylation of Smad2 and Stat6, respectively. Whereas treatment of primary microglia with TGFβ1 resulted in increased nuclear accumulation of Smad2/3, IL4 treatment failed to induce nuclear accumulation of Smad2/3 (Figure 5A). Immunoblotting against phospho-Smad2 and phospho-Stat6 revealed that TGFβ1 exclusively increased the levels of phosphorylated Smad2 and failed to increase the levels of phosphorylated Stat6. Vice versa, IL4 treatment resulted in increased levels of phospho-Stat6, whereas phosphorylation of Smad2 was not observed after treatment with IL4 for 1 and 2 hours (Figure 5B).

**Figure 6:**
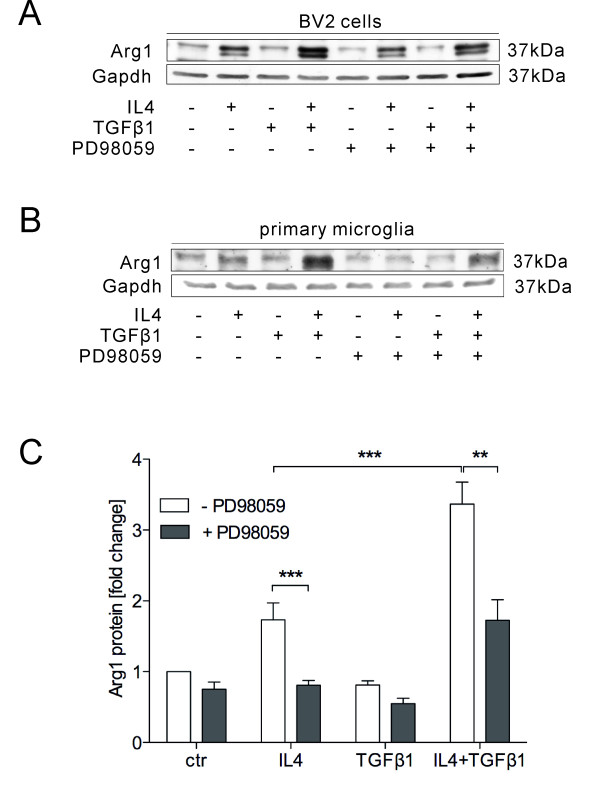
**TGFβ1-mediated enhancement of IL4-induced Arg1 expression is dependent on the mitogen-activated protein (MAP) kinase pathway.** BV2 cells and primary microglia were treated with IL4 (10 ng/ml), TGFβ1 (1 ng/ml) and IL4/TGFβ1 in the presence or absence of the MAP kinase inhibitor PD98059 (10 μM) for 24 hours. Total proteins were isolated and used for electrophoresis and western blotting. Arg1 protein levels were analysed by densitometric evaluation and normalised to GAPDH. The expression levels of Arg1 after co-treatment with IL4 and TGFβ1 were reduced in the presence of PD98059 in both BV2 (**A**) and primary microglia (**B**). Quantification of Arg1 expression levels in primary microglia after different treatments (**C**). Data are presented as means ± standard error from three independent experiments: **P < 0.01, ***P < 0.001 (two-way analysis of variance).

MAPK has been shown to be activated in microglia after TGFβ1 treatment [32]. To analyse the role of MAPK signalling on TGFβ1-mediated enhancement of IL4-induced Arg1 expression, BV2 cells and primary microglia were treated with IL4, TGFβ1 and IL4/TGFβ1 in the absence or presence of the MEK1/2 inhibitor PD98059 for 24 hours. Western blotting results from BV2 cells showed that IL4 treatment alone increased Arg1 protein levels, which was partially inhibited in the presence of PD98059. TGFβ1 and IL4 co-treatment increased IL4-induced Arg1 protein levels and the MEK1/2 inhibitor PD89059 partially blocked the TGFβ1-mediated increase in Arg1 protein levels (Figure 6A). Using primary microglia we confirmed the results obtained with BV2 cells. Treatment with IL4 significantly increased the protein levels of Arg1. Interestingly, in the presence of PD89059, IL4 failed to increase the protein levels of Arg1. Combination of IL4 and TGFβ1 dramatically increased the protein levels of Arg1 compared to IL4 treatment alone. However, in the presence of the MEK1/2 inhibitor PD89059, TGFβ1-enhanced Arg1 up regulation was significantly impaired and the amount of Arg1 was similar to the levels after treatment with IL4 alone (Figure 6B, C). These data demonstrate that TGFβ1-activated MAPK signalling is essential for TGFβ1-mediated enhancement of IL4-induced Arg1 expression in microglia.

## Discussion

In this study we demonstrate for the first time that TGFβ enhances the IL4-induced alternative activation of microglia. Using Arg1 and Ym1 as established markers for alternative activation [3,11] we provide evidence that IL4-mediated up-regulation of *Arg1* and *Ym1* is significantly enhanced in the presence of TGFβ1. Further, IL4 treatment resulted in increased expression and secretion of TGFβ2, whereas TGFβ treatment of microglia increased the expression of the IL4Rα. Moreover, blocking the TGF-β receptor type I resulted in significantly impaired Arg1 and Ym1 up-regulation after IL4 treatment. Finally, we demonstrate that TGFβ-mediated enhancement of Arg1 expression in microglia is dependent on the MAP kinase pathway.

In parallel to transcriptional regulation of microglia markers, the morphological changes are used to discriminate between different activation states *in vivo* and *in vitro*. In the resting or inactive state, microglia present a ramified morphology with several processes, while stimulation with classical activation factors such as LPS or IFNγ results in retraction of microglial processes and development of an amoeboid phenotype [33,34]. Although changes in morphology also suggest changes in the functional states of microglia, the morphology alone cannot be used to predict a functional outcome. Therefore, we analysed Arg1 and Ym1 as markers for macrophage and microglia alternative activation. Arg1 has been shown to be localised in the cytoplasm of hepatocytes where it is involved in nitrogen elimination by catalysing arginine hydrolysis to urea and ornithine [11,35]. Unlike the constitutively expressed Arg1 in the liver, Arg1 in macrophages and microglia is induced by exogenous stimuli including the Th2 cytokines IL4 and IL13 [36,37]. Arg1 inhibits NO production by competing with the inducible nitric oxide synthase (iNOS) for the common substrate L-arginine [38]. On the other hand, the production of ornithine can be used to generate polyamines, glutamate, and proline, the latter being a substrate for the formation of extracellular matrix proteins such as collagen [38-40]. Interestingly, apart from involvement in the regulation of wound healing and fibrosis [41,42], Arg1 can directly support neuron survival [43]. Next to Arg1, Ym1 is another established marker for microglia alternative activation [2,44]. Ym1 is a heparin/heparin sulphate-binding lectin that is transiently expressed during inflammation [44] and although the precise functions of Ym1 remain elusive, recent reports have suggested an involvement in tissue remodelling and regulation of inflammation [45,46].

TGFβ has been shown to either up-regulate Arg1 expression or increase the Arg1 enzymatic activity in a cell type-dependent manner. Whereas TGFβ treatment results in increased expression of Arg1 in fibroblasts and epithelial cells [47-49], TGFβ strongly increases enzyme activity in macrophages [50,51]. In this study we demonstrate that TGFβ1 alone is not able to significantly increase expression of Arg1 and Ym1 in microglia. However, in the presence of IL4, TGFβ1 significantly enhanced IL4-induced Arg1 and Ym1 expression, which indicates the potential role of TGFβ in CNS tissue repair and neurorestoration by modulating alternative activation of microglia.

To understand the mechanisms behind this phenomenon we addressed the question of whether TGFβ interferes with the IL4 signalling pathway. We demonstrated that TGFβ1 treatment alone up regulated the common receptor for IL4 and IL13, IL4Rα, both at mRNA and protein levels in a time-dependent manner. Opposite to the effect of TGFβ1 on IL4Rα expression, IL4 treatment alone reduced IL4Rα expression with treatment time (data not shown). Further, we observed that TGFβ1 was able to enhance the expression of Arg1 induced by IL13 (data not shown) and IL4. These data indicate that the synergistic effect of TGFβ1 and IL4 on the expression of Arg1 might partially be mediated by enhanced IL4Rα expression after TGFβ1 treatment, thereby increasing the sensitivity of microglia for IL4. IL4 signalling is further propagated by phosphorylation of the transcription factor Stat6 [11,31]. Analysis of Stat6 phosphorylation revealed that TGFβ1 failed to induce Stat6 phosphorylation. Moreover, IL4 was not able to induce Smad2 phosphorylation in microglia, indicating that the synergistic effect of TGFβ1 and IL4 on the expression of Arg1 could not be explained by the direct interaction of the TGFβ/Smad and IL4/Stat6 signalling pathways. Therefore, we further investigated if a TGFβ-induced Smad-independent pathway, the MAPK pathway, is involved in this synergistic effect. By performing a pharmaceutical blockage of the TGFβ-induced MAPK pathway using the MEK1/2 inhibitor PD98059, we could show that the Arg1 protein expression, not only induced by TGFβ1 and IL4 co-treatment but also induced by IL4 treatment alone, were significantly inhibited in the presence of PD98059. These data clearly demonstrate that TGFβ signalling is involved in IL4-induced microglia alternative activation and an essential role of TGFβ-mediated MAPK pathway in the enhancement of IL-4 induced microglia alternative activation by TGFβ signalling.

We observed that IL4 treatment of microglia lead to up regulation of TGFβ2, whereas the mRNA levels of TGFβ1 and TGFβ3 were not changed after IL4 treatment. TGFβ2 levels were significantly increased in the supernatants of IL4-treated microglia. Although most of the secreted TGFβ2 was in a latent and inactive form, a small proportion of bioactive TGFβ2 seems to be sufficient to support IL4-induced up regulation of Arg1 and Ym1. However, microglia express several factors and enzymes that are capable of activating latent TGFβs, such as integrins, plasminogen, MMP2 and thrombospondin-1 (unpublished data). Moreover, microglia also express extracellular matrix components *in vitro* that might bind TGFβ. This amount of bound, and probably activated, TGFβ will escape all analyses of the microglial supernatant, but is likely to activate TGFβ signalling in these cells.

It is widely accepted that TGFβ is involved in the down regulation of microglia classical activation. TGFβ1 reduces reactive oxygen species (ROS) induced by LPS and suppresses the IFNγ-induced expression of MHC II and the production of cytokines, IL1, IL6, and TNF-alpha production in activated microglia [52,53]. TGFβ also prevents IL1β-induced microglial activation [54]. Although the anti-inflammatory role of TGFβ has been widely accepted, it is still quite ambiguous whether this effect is beneficial or detrimental in terms of different CNS diseases. Whereas TGFβ1 has protective and beneficial functions in cerebral ischaemia [55], it promotes the deposition of amyloid-beta plaques in models of Alzheimer’s disease [56]. Interestingly, Town and colleagues have demonstrated that blocking of TGFβ/Smad signalling almost completely abrogated the plaque formation in transgenic mice overexpressing mutant human amyloid precursor protein [57]. These results underline the importance of a tight temporal and spatial regulation of innate immune responses and further demonstrate the necessity to enhance our knowledge of the pathological conditions under which TGFβ-mediated regulation of inflammation is beneficial or detrimental.

Whereas TGFβ induces acquired deactivation, the acquired deactivation macrophages also produce TGFβ in an autocrine manner [3,24,25]. Next to down regulating the classical activation of microglia, here we show, for the first time, that the TGFβ also enhances IL4-induced microglia alternative activation *in vitro*, which broadens the knowledge of interactions among different microglia activation states. Similar functions have been shown for another immunoregulatory cytokine, IL10. For example, IL10 is able to impair IFNγ-induced macrophage classical activation [58], increase arginase activities [59], and further enhance IL4-induced Arg1 expression, probably by increasing IL4Rα expression [60]. Findings of this work and previous studies suggest an interaction and dynamic change between different microglia activation states. TGFβ might serve as a gatekeeper to inhibit classical activation and promote alternative activation of microglia. The data presented throughout this study confirm the role of TGFβ as an anti-inflammatory molecule and broaden its functions as an enhancer of microglia alternative activation, thereby regulating microglia-mediated neuroregeneration and neurorestoration in inflammatory CNS diseases.

## Conclusions

**Figure 7:**
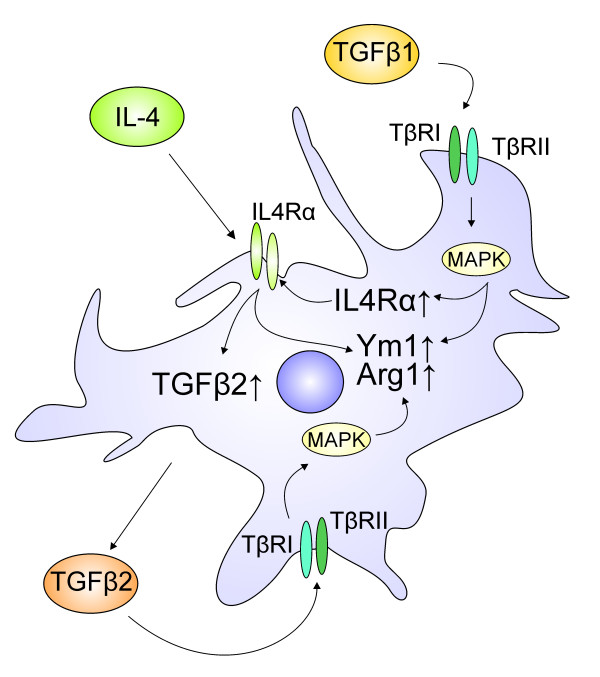
**Proposed model for the role of TGFβ in IL4-induced alternative microglia activation.** IL4 induces expression of the alternative activation markers Ym1 and Arg1 via the IL4Rα-Stat6 pathway. TGFβ binds to its receptors TGFβ type I and type II, which form a heteromeric complex and initiate Smad-dependent and Smad-independent pathways. Exogenous TGFβ1 enhances IL4-induced Ym1 and Arg1 expression either by a direct effect on Ym1/Arg1 promoter activity or indirectly by up regulating the IL4Rα through activation of the MAP kinase (Smad-independent) pathway. Furthermore, IL4 treatment alone increased endogenous TGFβ2 expression and secretion. Autocrine TGFβ2 in turn might be able to enhance IL4-induced Arg1 expression by using similar signalling mechanisms to exogenous TGFβ1.

Here we show, for the first time, that TGFβ1 synergises IL4 in the induction of microglia alternative activation. We demonstrate that IL4 treatment increased the expression and secretion of TGFβ2 in primary microglia and that IL4-induced up regulation of Arg1 and Ym1 is dependent on active TGFβ signalling. Finally, we provide evidence that MAPK signalling is involved in TGFβ-mediated enhancement of IL4-induced microglia alternative activation. Figure 7 shows a proposed model for the role of TGFβ in microglia alternative activation. Our findings provide novel insights into the molecular mechanisms of IL4-induced microglia alternative activation, and further enhance our knowledge of TGFβ-mediated modulation of microglial functions.

## Abbreviation

AD: Alzheimer’s disease; Arg1: Arginase1; Ym1: Chitinase 3-like 3; CNS: Central nervous system; DAPI: 4′,6-diamidino-2-phenylindole; DMEM: Dubecco’s modified Eagle medium; ELISA: Enzyme-linked immunosorbent assay; FBS: Fetal bovine serum; FCS: Fetal calf serum; GAPDH: Glyceraldehyde-3-phosphate dehydrogenase; LPS: Lipopolysaccharide; M1: Classical activation of microglia; M2: Alternative activation of microglia; MAPK: Mitogen-activated protein kinase; MCM: Microglial conditioned medium; MLEC: Mink lung epithelial cell; MMP: Matrix metalloproteinase; M-PER: Mammalian protein extraction reagent; MS: Multiple sclerosis; NO: Nitric oxide; P/S: Penicillin/streptomycin; PAI: Plasminogen activator inhibitor; PBS: Phosphate-buffered saline; PD: Parkinson’s disease; PFA: Paraformaldehyde; PVDF: Polyvinylidene difluoride; ROS: Reactive oxygen/nitrogen species; TNFα: Tumour necrosis factor-alpha.

## Competing interests

The authors declare that they have no competing interests.

## Authors’ contributions

XZ conceived of the study idea. XZ carried out all the experiments. BS participated in the experiments. XZ, BS and KK were involved in conception and design of the study as well as in the manuscript preparation. All authors have read and approved the final manuscript.
